# Opposing Actions of TLR2 and TLR4 in Adipocyte Differentiation and Mature-Onset Obesity

**DOI:** 10.3390/ijms232415682

**Published:** 2022-12-10

**Authors:** Natalia Cuesta, Sonia Fernández-Veledo, Carmen Punzón, Cristóbal Moreno, Beatriz Barrocal, Vinatha Sreeramkumar, Manuel Desco, Manuel Fresno

**Affiliations:** 1 School of Medicine, Universidad Complutense de Madrid, 28040 Madrid, Spain; 2 Instituto de Investigación Sanitaria Pere Virgili, University Hospital of Tarragona Joan XXIII, 43007 Tarragona, Spain; 3 Centro de Biología Molecular Severo Ochoa, Universidad Autónoma de Madrid—Consejo Superior de Investigaciones Científicas, 28049 Madrid, Spain; 4 School of Medicine, Universidad Alfonso X el Sabio, Villanueva de la Cañada, 28691 Madrid, Spain; 5 School of Health and Biomedical Sciences, Universidad Europea de Madrid, Villaviciosa de Odón, 28670 Madrid, Spain; 6 Department of Bioengineering and Aerospace Engineering, Universidad Carlos III de Madrid, 28911 Leganés, Spain

**Keywords:** adipocyte differentiation, TLR2, TLR4, obesity

## Abstract

Understanding the signaling cascades that govern adipocyte metabolism and differentiation is necessary for the development of therapies for obesity. Toll-like receptors (TLRs) are key mediators in adipogenesis, but their specific role is not completely understood. In this study, siRNA knockdown of *Tlr2* in 3T3-L1 cells allowed them to differentiate more efficiently into adipocytes, whereas the opposite was observed for the knockdown of *Tlr4*. At the same time, we show that TLR2 knock-out mice spontaneously developed mature-onset obesity and insulin resistance. Besides a higher incidence of hyperplasia and hypertrophy in white adipose tissue (WAT), we found a significantly increased number of adipocyte precursor cells in TLR2^−/−^ mice compared to TLR4^−/−^ mice. Interestingly, genetic inactivation of *Tlr4* in TLR2^−/−^ mice reverted their increased adiposity, insulin resistance, and restored normal levels of adipocyte precursor cells. These findings provide evidence that TLR2 and TLR4 play opposing roles in WAT homeostasis and point to the existence of cross-regulation among TLR2 and TLR4 during adipocyte differentiation both in vitro and in vivo.

## 1. Introduction

The obesity epidemic is currently developing into a major health problem of industrialized countries. Obesity has been related to the development of cardiovascular diseases, type 2 diabetes mellitus, fatty liver disease, and some cancers [1,2,3]. However, treatment has proven to be difficult, given the multiple factors involved in the development of the disease [1,4,5]. Thus, the knowledge of factors inducing obesity may open new possibilities for therapy.

The increase in adipose tissue during obesity is the result of both hypertrophy and hyperplasia of adipocytes [6], but it also depends on the generation of new adipocytes from progenitor cells resident in the WAT [7]. This population has been characterized by the expression of several surface markers: Lin^−^:CD29^+^:CD34^+^:Sca-1^+^:CD24^+^ [8]. Adipogenic differentiation is a tightly regulated process involving the expression of many adipose-specific genes necessary for lipid transport and synthesis, insulin sensitivity, and the secretion of adipocyte-specific proteins [9].

Obesity is now viewed as a state of chronic low-grade inflammation [10,11]. Moreover, inflammatory signaling cascades activated during mature-onset obesity have been linked to the development of insulin resistance [12,13]. Insulin resistance is characterized by a failure to respond properly to normal levels of circulating insulin in several tissues. The earliest abnormality observed in insulin resistance is a decrease in insulin-induced glucose uptake in skeletal muscle and adipose tissue [14]. The impairment of insulin sensitivity involves multiple organs, but prominently includes hypertrophic adipose tissue with an associated rise in serum and tissue levels of adipokines and proinflammatory molecules such as interleukin-6 (IL-6) and tumor necrosis factor-alpha (TNF-α) [15].

Recently, Toll-like receptors (TLRs) have been added to the list of molecules relevant to the development of obesity and diabetes (reviewed in [16,17,18,19]). TLRs are expressed by adipocytes [20,21,22] and immune system cells, positioning them in the crossroads between obesity and inflammation [19,23]. TLRs are pattern recognition receptors that recognize microbial products that are conserved among pathogens [24], and they are also capable of recognizing a greater variety of ligands. Among those, both TLR2 and TLR4 can respond to nutritional fatty acids [25,26,27,28,29]. However, the studies that tried to elucidate their specific role in obesity did not follow parallel paths.

Several studies originally showed that TLR4 deficiency prevented diet-induced obesity (DIO) and insulin resistance in mice [30,31,32,33]. It was later found that both saturated fatty acids and the hormone resistin bind TLR4 and mediate lipid overload-induced inflammation and metabolic diseases [34,35]. However, some controversy exists regarding TLR2. Whereas some groups have reported that inhibiting TLR2 confers protection to insulin resistance and DIO in mice [36,37,38,39], other studies point to the exact opposite [40].

Other than the animal models, in vitro experiments using the 3T3-L1 cell line have sought to investigate the role of TLR2 and TLR4 in adipocyte differentiation. 3T3-L1 cells are multipotent mouse fibroblasts that can differentiate into adipocyte-like cells and are widely used as a common in vitro model for adipocyte differentiation [41]. Thanks to these studies, we know that TLR4 is induced during adipocyte differentiation and its expression is enhanced following fatty acid stimulation [27], whereas TLR2 and TLR6 expression is constant throughout the differentiation of 3T3-L1 cells [22]. Silencing TLR4 offered protection from saturated fatty acids-induced inflammation in vitro [30], and TLR2/TLR1 activation was able to interfere with adipocyte differentiation in the 3T3-L1 cell line [22].

Taking all this into account, we sought to determine the specific role of TLR2 and TLR4 in adipocyte differentiation by blocking the expression of *Tlr2* and *Tlr4* in 3T3-L1 cells with siRNA.

We also studied the role of TLR2 and TLR4 in the development of mature onset obesity and insulin resistance by using TLR2^−/−^ and TLR4^−/−^, as well as TLR2^−/−^-TLR4^−/−^ mice. Our findings prove that TLR2 negatively regulates adipocyte differentiation and that TLR2 and TLR4 play opposing roles in the development of mature onset obesity in mice.

## 2. Results

### 2.1. Tlr2, but Not Tlr4, Negatively Regulates Adipogenic Differentiation In Vitro

**Figure 1:**
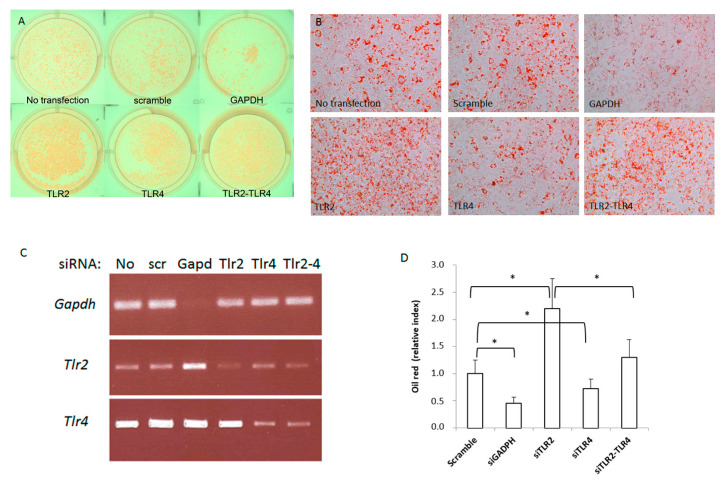
Silencing *Tlr2* or *Tlr4* gene expression in 3T3-L1 cells results in different adipogenic differentiation outcomes. 3T3-L1 cells were induced to differentiate after siRNA transfection of a scrambled siRNA or specific siRNAs for *Gapdh*, *Tlr4* and *Tlr2* or both *Tlr2* and *Tlr4*. (**A**) Plate photograph of intracellular lipids in 3T3−L1 cells stained with Oil red O solution. Pictures were taken 5 days after induction of adipogenesis; a representative picture is shown. (**B**) 3T3-L1 cell monolayers were photographed with an inverse phase contrast microscope at a final magnification of 200×. (**C**) Efficiency of gene expression silencing analysed by RT-PCR (No: no transfection; scr: scramble). (**D**) Quantification of oil red in three independent experiments performed in duplicate (* *p* < 0.05). Results are expressed as the mean ± standard deviation.

To test the specific role of TLR2 and TLR4 in adipocyte differentiation, we first studied the effect of silencing *Tlr2* and/or *Tlr4* in the 3T3-L1 cell line (Figure 1). 3T3-L1 cells were induced to differentiate after siRNA transfection of a scrambled siRNA (negative control) or specific siRNAs for *Gapdh* (positive control), *Tlr4* and *Tlr2* or both *Tlr2* and *Tlr4.* At day 5 after siRNA transfection and induction of differentiation, cells were stained for lipid accumulation using Oil Red O (Figure 1A,B). Then, 48 h after transfection, total RNA was isolated and the efficiency of knocking-down the expression of these genes was assessed by RT-PCR. Although the silencing was not complete, we observed less than 50% of expression of the corresponding mRNAs in each case (Figure 1C). 3T3-L1 cells transfected with siRNA for *Gapdh* showed reduced ability to differentiate into adipocytes, in contrast with scrambled siRNA transfected cells. Interestingly, inhibition of *Tlr2* expression by siRNA enhanced adipocyte differentiation, as seen by the accumulation of lipid droplets and the Oil red O dye. In contrast, inhibition of *Tlr4* decreased differentiation. Far more intriguing, silencing of both *Tlr4* and *Tlr2* resulted in the same phenotype as the negative control (Figure 1A,B).

### 2.2. TLR2^−/−^ Mice but Not TLR4^−/−^ Mice Develop Mature-Onset Obesity

**Figure 2:**
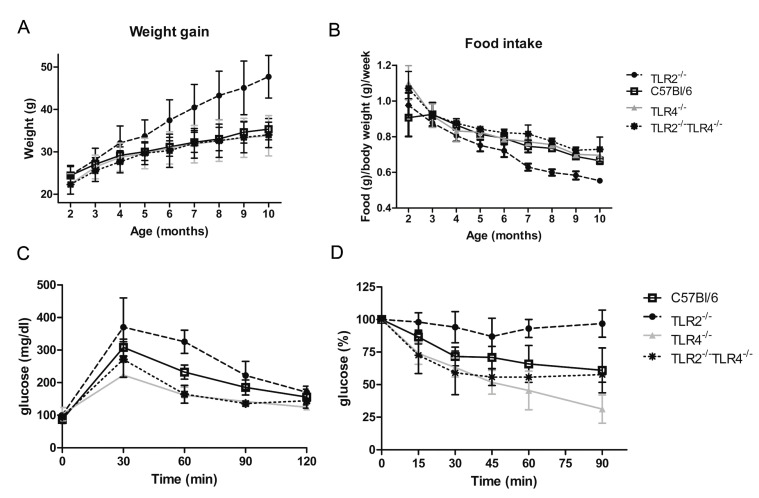
Loss of function of TLR4 reverses the obesity phenotype of TLR2^−/−^ mice. (**A**) Evolution of body weight in TLR2^−/−^, TLR4^−/−^, TLR2^−/−^-TLR4^−/−^ and WT mice (*n* = 40 mice per group; *p* < 0.001 between TLR2^−/−^ mice and WT mice starting at 4 months and continuing till 10 months; no significant differences between the other groups of mice). (**B**) Food intake of TLR2^−/−^, TLR4^−/−^, TLR2^−/−^-TLR4^−/−^ and WT mice (n = 40 mice per group; *p* < 0.001 between TLR2^−/−^ mice and WT mice starting at 3 months and continuing till 10 months; no significant differences between the other groups of mice). (**C**) GTT of TLR2^−/−^, TLR4^−/−^, TLR2^−/−^-TLR4^−/−^ and WT mice at 7 months of age (n = 5 per group; *p* < 0.01 between TLR2^−/−^ mice and WT mice at 60 min; *p* < 0.05 between TLR4^−/−^ mice and WT mice at 30 and 60 min; no significant differences between TLR2^−/−^-TLR4^−/−^ and WT mice). (**D**) ITT of TLR2^−/−^, TLR4^−/−^, TLR2^−/−^-TLR4^−/−^ and WT mice at 7 months of age (n = 5 per group; *p* < 0.001 between TLR2^−/−^ mice and WT mice at 90 min; *p* < 0.05 between TLR2^−/−^ mice and WT mice at 30 and 60 min; *p* < 0.01 between TLR4^−/−^ mice and WT mice at 90 min; no significant differences between TLR2^−/−^-TLR4^−/−^ and WT mice). Values are represented as mean ± s.d.

As *Tlr2* and *Tlr4* deficiency showed opposite effects on adipocyte differentiation, we analysed more in detail the role of those deficiencies in vivo. TLR2^−/−^, TLR4^−/−^, and WT male mice placed on a normal diet were monitored for 40 weeks. TLR2^−/−^ mice increased body weight at a significantly higher rate than WT mice after 3 months of age (Figure 2A). However, this increase in body weight was not accompanied by an increase in food intake. In fact, food consumption per body weight was significantly lower in TLR2^−/−^ mice than in WT mice (Figure 2B). In contrast to TLR2^−/−^ mice, TLR4^−/−^ and WT mice showed comparable body weight gain and caloric intake (Figure 2A,B). To assess whole-body glucose homeostasis, we next performed intraperitoneal glucose tolerance tests (GTT) and insulin tolerance (ITT) tests. TLR2^−/−^ mice demonstrated marked glucose intolerance and were also highly resistant to the hypoglycaemic effect of exogenous insulin at 7 months of age. Despite no significant differences in age-onset obesity, TLR4^−/−^ mice cleared blood glucose more efficiently than WT littermates in both a GTT (Figure 2C) and an ITT (Figure 2D) at 7 months of age. This indicates that the glucose utilization rate of TLR4^−/−^ mice is higher than in WT mice, and thus, TLR4^−/−^ mice are more sensitive to insulin than WT.

### 2.3. Loss of Function of TLR4 Reverses the Obesity Phenotype of TLR2^−/−^ Mice

Given that TLR2^−/−^ and TLR4^−/−^ mice showed an opposite phenotype, and that TLR4 deficiency reversed TLR2 deficiency phenotype in 3T3L1 cells, we wondered whether knocking-down *Tlr4* in TLR2^−/−^ mice would reverse their adiposity and insulin resistant phenotype. As shown in Figure 2A, the body weight curve of TLR2^−/−^-TLR4^−/−^ mice resembled that of TLR4^−/−^ mice, indicating that the obesity developed by TLR2^−/−^ mice depends on the expression of TLR4. In addition, the capability of TLR2^−/−^-TLR4^−/−^ mice to clear glucose from blood after a GTT and an ITT test is similar to TLR4^−/−^ mice (Figure 2C,D). In summary, genetic inactivation of *Tlr4* in TLR2^−/−^ mice reverts their increased adiposity and insulin resistance.

### 2.4. Loss of Function of TLR2 Is Associated with Fat Accumulation in Mice

**Figure 3:**
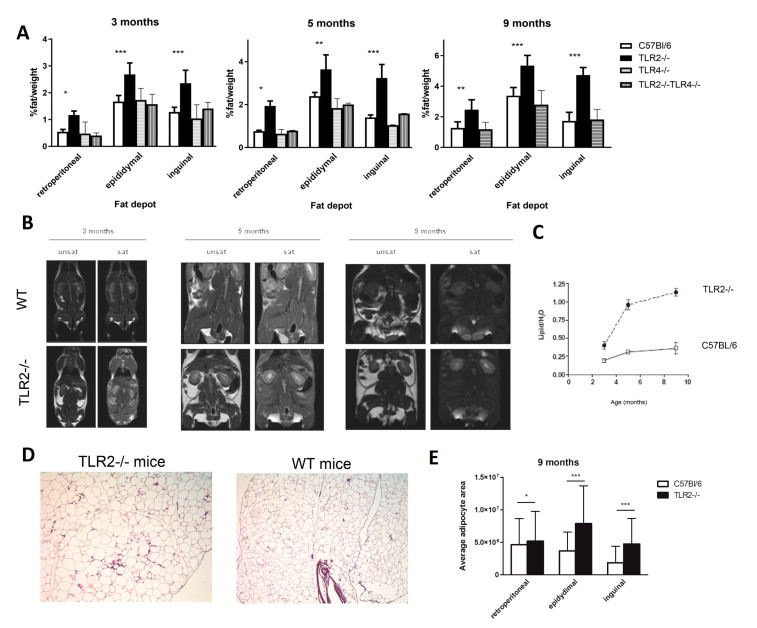
TLR2^−/−^ mice develop excess adiposity. (**A**) Relative weights of different fat depots isolated from TLR2^−/−^, TLR4^−/−^, TLR2^−/−^-TLR4^−/−^, and WT mice. The relative fat mass was expressed as a percentage of body weight (n = 4, two-way ANOVA, * *p <* 0.05, ** *p <* 0.01, *** *p <* 0.001 between TLR2^−/−^ mice and WT mice). Values are represented as mean ± s.d. (**B**) NMR images of whole-body horizontal sections were taken at the approximate midline. White density denotes fat. (**C**) Quantification of lipid in WAT based on spectroscopy of lipid and water peaks. (n = 3 per group, two-way ANOVA). Values are represented as mean ± s.d. (**D**) Histology of the epidydimal WAT of TLR2^−/−^ and WT mice (hematoxylin-eosin staining; scale bar, 200 μm). A representative picture is shown at 7 months of age (**E**) Average adipocyte area measured in histology sections from different fat depots of TLR2^−/−^ and WT mice. (n = 3 per group, two-way ANOVA, * *p <* 0.05, *** *p <* 0.001). Values are represented as mean ± s.d.

Since only TLR2^−/−^ mice showed a clear mature-onset obese phenotype, we analysed the underlying mechanism in more detail. This phenotype was accompanied by enlarged epididymal, inguinal, and retroperitoneal fat deposits, which were already significant at 3 months of age, when still no significant differences in weight were observed. The percentage of fat weight related to the body weight was significantly higher in TLR2^−/−^ mice than in the other strains (Figure 3A). NMR analysis showed a markedly increased percentage of fat mass in TLR2^−/−^ mice (Figure 3B). TLR2^−/−^ mice already had a 3-fold increase in the percentage of lipid to water ratio at 5 months of age, and this increase was sustained until the age of 9 months (Figure 3C).

Morphometric analysis revealed that adipocytes were significantly larger in WAT from TLR2^−/−^ mice than in WT counterparts (Figure 3D,E). These results indicate that loss of function of TLR2 is associated with fat accumulation as early as 3 months of age in mice.

### 2.5. Leptin, MCP-1, and TNF-α Levels Are Increased in the Serum of TLR2^−/−^ Mice

Serum TNF-α levels were significantly higher in TLR2^−/−^ mice than in WT littermates at 5 months and 9 months of age, whereas serum IL-10 levels were significantly lower at 5 months. No significant differences were found in the levels of these pro-inflammatory cytokines between the other mouse strains (Supplementary Figure S1A,B). As expected from increased WAT mass, serum leptin concentrations were higher in TLR2^−/−^ mice at 5 months and 9 months of age. In contrast, serum adiponectin levels, an insulin-sensitizing adipokine, remained unchanged (Supplementary Figure S1A,B). Finally, serum levels of MCP-1 were found to be significantly increased in TLR2^−/−^ mice at both 5 and 9 months of age (Supplementary Figure S1A,B).

### 2.6. TLR2^−/−^ Mice Show Altered Metabolic Profiles

We then studied the lipid metabolism and found that circulating saturated fatty acids (FAs) were increased by two-fold in 9-month-old TLR2^−/−^ mice when compared to WT mice. On the contrary, desaturated fatty acids (FAd) were decreased (Supplementary Figure S2A). Fasting serum triglyceride (TG) levels of TLR2^−/−^ mice were also increased at 9 months of age (Supplementary Figure S2B). We next studied adipose tissue lipolysis efficiency by measuring plasma nonesterified fatty acids (NEFA) and glycerol levels in both fasting and fed conditions. No significant differences were found between the two strains (Supplementary Figure S2C,D), indicating that adipose tissue lipolysis was not affected by the loss of function of TLR2.

### 2.7. TLR2^−/−^ Mice Develop Insulin Resistance

**Figure 4:**
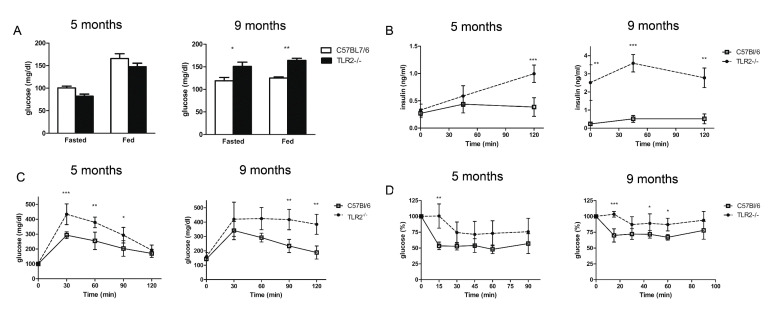
TLR2^−/−^ mice develop insulin resistance. *(***A**) Serum concentrations of glucose in TLR2^−/−^ vs. WT mice at 5 and 9 months of age during the fed and fasted states. (**B**) GTT of TLR2^−/−^ vs. WT mice at 5 and 9 months of age. (**C**) Serum insulin levels during the glucose tolerance test (n = 5 per group, two-way ANOVA). (**D**) ITT of TLR2^−/−^ vs. WT mice at 5 and 9 months of age (n = 5 per group, two-way ANOVA, * *p <* 0.05, ** *p <* 0.01, *** *p <* 0.001). Values are represented as mean ± s.d.

Given the excess of lipid deposition in the WAT of TLR2^−/−^ mice, we decided to examine blood glucose and insulin levels. Both TLR2^−/−^ and WT mice exhibited similar fasting levels of glucose at 5 months of age, but TLR2^−/−^ mice showed elevated blood glucose levels at 9 months (Figure 4A). Hyperglycaemia in TLR2^−/−^ mice occurred despite a marked increase in basal serum insulin levels at 9 months of age (Figure 4B), indicating that TLR2^−/−^ mice were becoming more resistant to insulin than their WT counterparts with age. The GTT study demonstrated marked glucose intolerance in TLR2^−/−^ mice that developed as early as at 5 months of age (Figure 4C). During the GTT, serum insulin levels increased and were significantly higher in 5-month-old TLR2^−/−^ mice 30 min after glucose injection and they continued increasing for the next 90 min. Moreover, in 9-month-old animals, serum insulin levels were much higher at all time points during the GTT in TLR2^−/−^ mice (Figure 4B). The ITTs showed that TLR2^−/−^ mice were also highly resistant to the hypoglycaemic effect of exogenous insulin both at 5 and 9 months of age (Figure 4D).

### 2.8. Absence of TLR2 Correlates with an Increase in mRNA Gene Expression for Adipogenesis-Related Genes in WAT

**Figure 5:**
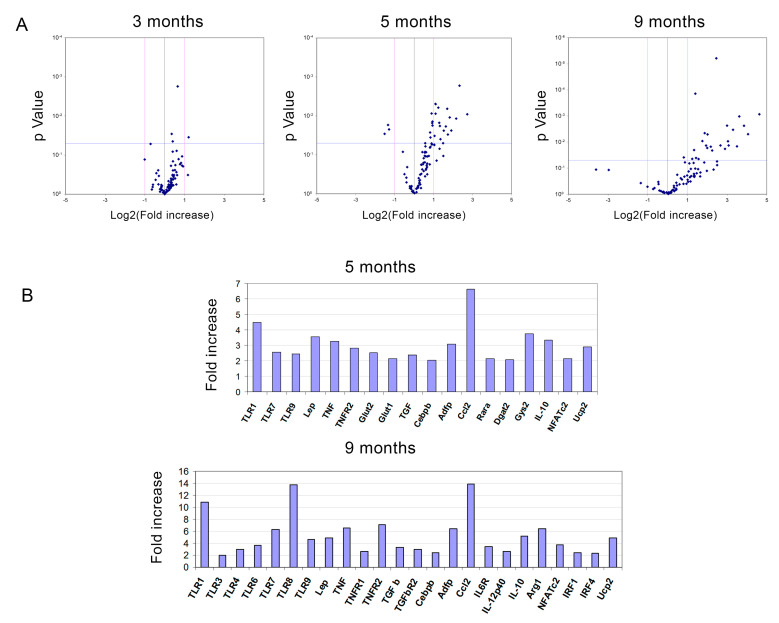
Loss of TLR2 correlates with an increase in mRNA gene expression of adipogenesis-related genes in WAT. (**A**) Volcano plots of gene expression data in epididymal WAT comparing P values versus fold change plotted for TLR2^−/−^ vs. WT mice at 3, 5, and 9 months of age. (**B**) Bar graphs showing the significantly up-regulated genes in the WAT of TLR2^−/−^ mice at 5 and 9 months of age. The genes were detected by real-time PCR in microfluidic cards.

To obtain further insight into the underlying mechanism, mRNA from several genes associated to obesity was analysed in epididymal fat from both TLR2^−/−^ and WT mice at 3, 5, and 9 months of age (Figure 5). The study showed an increase in the expression of some adipogenic genes in the WAT of TLR2^−/−^ mice. Only *Gys2* was shown to be up-regulated more than 2-fold in the WAT of 3-month-old TLR2^−/−^ mice when compared to WT. Glycogen synthase 2 (Gys-2) is the rate-limiting enzyme for glycogen synthesis in liver and adipose tissue. An increase in the activity of this enzyme has been correlated to enhanced lipogenesis and TG storage in adipose tissue [42]. As the TLR2^−/−^ mice grew older, increased expression of genes involved in lipid accumulation in adipocytes (*Gys2*, *Dgat2* and *Adfp*) and transcription factors necessary for the differentiation of adipocytes (*Cebpb*) were observed (Figure 5). Increased gene expression was also documented for some cytokines (*Tnf*, *Tgfb1, IL-12b,* and *Ccl2*).

### 2.9. Fat Accumulation in TLR2^−/−^ Mice Is Associated with a Higher Number of Adipocyte Precursors in WAT and Is Reversed by TLR4 Deficiency

**Figure 6:**
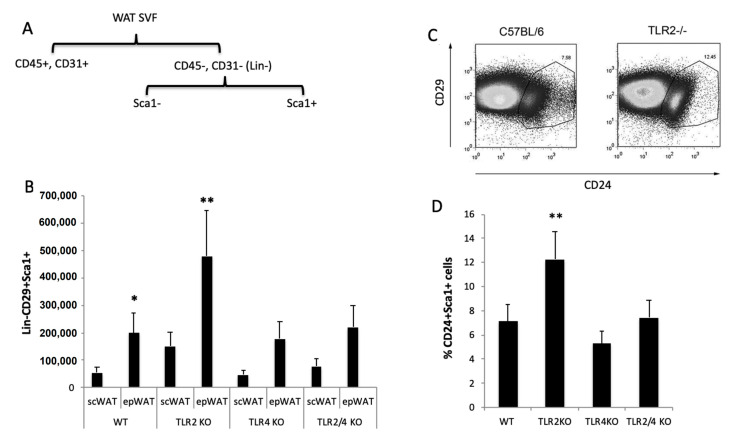
Higher number of adipocyte precursor cells in the WAT of TLR2^−/−^ mice. (**A**) Scheme of the purification of adipocyte precursors. (**B**) Total numbers of FITC-Sca-1^+^ cells stained with anti–CD29 in the epididymal and subcutaneous SVF of WT and TLR2^−/−^, TLR4^−/−^, and TLR2^−/−^-TLR4^−/−^ mice. Average data from three experiments of three animals per group and performed in duplicate. Values are represented as mean + s.d. (* *p <* 0.05, ** *p <* 0.01) (**C**) FACS plots analysing the populations of adipocyte precursor cells in the epididymal WAT SVF of TLR2^−/−^ vs. WT mice. Sorted CD29^+^Sca1^+^ cells were stained with CD24 to identify early adipocyte progenitor cells. One representative FACS experiment of four experiments performed in duplicate is plotted. (**D**) Percentage of CD24^+^ cells in the Lin^−^CD29^+^:CD34^+^:Sca-1^+^ cell population in the SVF of the epididymal WAT of WT, TLR2^−/−^, TLR4^−/−^ and TLR2^−/−^-TLR4^−/−^ animals. Average data from three experiments of three animals per group and performed in duplicate. Values are represented as mean + s.d. (** *p <* 0.01).

To determine the number of adipocyte progenitor cells, we employed magnetic separation to discriminate WAT cell populations from freshly isolated stromal vascular fraction (SVF). After depletion of endothelial cells and leukocytes, the remaining cells were selected for the expression of Sca-1 (Figure 6A). All the Sca-1^+^ cells were also positive for CD29 and CD34. As shown in Figure 6B, the absolute number of Lin^−^CD29^+^Sca1^+^ cells was significantly higher in TLR2^−/−^ mice, especially in epididymal but also in subcutaneous WAT. Interestingly the percentage of Lin^−^CD29^+^:CD34^+^:Sca-1^+^:CD24^+^ cells, which represent the early adipocyte progenitor cell population [25], was higher in the SVF of TLR2^−/−^ mice than in WT mice (an average of 12.25% vs. 7.15%, respectively) (Figure 6C,D). Thus, the total number of Sca1^+^CD24^+^ early adipocyte precursor cells in both epididymal and subcutaneous WAT from TLR2^−/−^ mice was found to be on average 3.5–4-fold higher than in WT mice. In contrast, similar analysis of TLR4^−/−^ WAT, show a slight decrease (not significant) in the Lin^−^CD29^+^:CD34^+^:Sca-1^+^ total cell number (Figure 6C) and in the percentages of Lin^−^CD29^+^:CD34^+^:Sca-1^+^:CD24^+^ cells (Figure 6D). More interestingly, knockdown of *Tlr4* in TLR2^−/−^ mice reverted its pro-adipocyte phenotype, resulting in a close-to-normal cell number and early adipocyte progenitor cell populations in SVF (Figure 6C,D).

### 2.10. The Number of Macrophages Infiltrating WAT Is Higher in TLR2^−/−^ Mice

To quantitate the proportion of macrophages (F4/80^+^ cells) within WAT, we prepared SVFs from TLR2^−/−^, TLR4^−/−^, TLR2^−/−^-TLR4^−/−^, and WT mice and analyzed them by flow cytometry. We found a higher percentage of F4/80^+^ cells in TLR2^−/−^ mice than in WT controls (22.01% of F4/80+ cells in TLR2^−/−^ mice vs. 15.21% in WT mice; Supplementary Figure S3). There were no significant differences between the other strains of mice.

## 3. Discussion

Recently, TLRs have been added to the list of molecules relevant to the development of obesity and diabetes [17,18,19]. The expression of TLR2 and TLR4 was described in adipocytes in 2000 [43], providing the first hint for a role of TLRs in adipose tissue. Results to date have found that all TLRs are expressed in adipose-tissue-derived mesenchymal stem cells, stromal vascular cells, and in different fat depots [20,44]. TLR2 and TLR4 were proven to be functional in mice and human, as isolated adipocytes and pre-adipocytes respond to specific stimulation by their ligands [20,45]. Moreover, TLR expression in mouse and human adipose tissue varies according to the differentiation state [20,46]. However, there are not enough studies focusing on the role of TLRs on adipocyte differentiation. With respect to TLR2, Poulain-Godefroy et al. showed in 2010 that exposure to the TLR2 ligand Pam3CSK4 impaired 3T3-L1 differentiation [11]. Following this line of evidence, our results show that knocking-down *Tlr2* in 3T3-L1 cells allows them to differentiate more efficiently into adipocytes. In addition, our results establish for the first time that TLR2 and TLR4 are crucial but opposite regulators of adipocyte differentiation, since blocking the expression of both *Tlr2* and *Tlr4* in 3T3-L1 cells reverts to the original phenotype.

Many studies indicate that TLR2 and TLR4 expression is increased in obesity and that global deficiency of TLR2 and TLR4 in DIO animal models ameliorates inflammation, insulin sensitivity, and weight gain, pointing to a pathogenic role of TLRs in obesity and insulin resistance via the induction of inflammation–although some discrepancies exist [47]. Our studies in mice show that TLR2 plays a fundamental role in the regulation of adipocyte differentiation and hypertrophy, insulin resistance, and glucose tolerance under normal diet conditions. Several authors have already reported the use of TLR2^−/−^ mice in obesity studies with controversial results. Many studies claimed that TLR2^−/−^ mice were substantially protected from diet-induced adiposity and insulin resistance [36,37,38,39]. These authors reported that plasma glucose and insulin levels were lower in TLR2^−/−^ mice, and that glucose tolerance and insulin sensitivity were increased in TLR2^−/−^ mice. However, one study was undertaken with much younger individuals (1–2 months old) [36]. These data are crucial for understanding WAT homeostasis. In fact, we obtain similar results with 3-month-old animals and start seeing significant differences in the glucose-lowering effects of insulin between the strains only when mice reach at least 5 months of age. Different diet compositions may also explain some discrepancies. We have employed a chow diet, whereas some reports used experimental high-fat diet compositions [36,37,39]. Kou et al. used the same animal model with a similar approach as ours but did not observe any difference in weight gain between TLR2^−/−^ and WT mice when they were fed a standard chow diet [38]. We must highlight some differences between our studies: Kou et al.’s diet contained meat and fish meal, whereas our diet was vegetarian. They also used females in their study, whereas we used males. We have excluded the possibility of hormonal changes interfering with our results by using only male mice and feeding them with a vegetarian diet. Further, we have used a minimum of 40 mice per group in the weight gain studies to avoid differences due to variations in the susceptibility to obesity that always appear in animal studies, and Kou et al. used seven mice per group to study body weights. Moreover, our findings in TLR2^−/−^ mice are further substantiated by the in vitro assays in 3T3-L1 cells, and correlate with the studies reported by Shechter et al. [40].

On the other hand, our observations in TLR4^−/−^ mice do correlate with previously published results showing that loss of function of TLR4 partly prevented diet-induced insulin resistance in mice and selectively protected against obesity [30,31,32,33]. A very remarkable result of our study is the reversion of the mature-onset obesity phenotype of TLR2^−/−^ mice by the generation of TLR2^−/−^TLR4^−/−^ double knockout mice. Similar results were observed in *Tlr2*-silenced 3T3L1 cells when *Tlr4* was also silenced. This points to the opposing role of both receptors in adipogenesis. In fact, TLR2^−/−^TLR4^−/−^ mice are undistinguishable from WT mice with respect to insulin resistance and obesity phenotypes. TLR2 and TLR4 have been demonstrated to act in a similar way in many systems [48], while at the same time, they play opposing roles in some others [49,50,51]. Similarly, TLR2 and TLR4 might be recognising specific ligands in adipose tissue, which might differentially activate signalling pathways leading to opposite responses.

The increased body weight observed in our study in TLR2^−/−^ mice can be explained in part by an increase in the number of adipocyte precursor cells found in the WAT with respect to WT mice. This finding is in line with previously published results that established that TLR2 activation reduced bone-marrow mesenchymal stem cell differentiation into the three mesodermal lineages [52]. Loss of TLR2 would then favour differentiation of mesenchymal precursors, which, accompanied by a higher number of precursor cells resident in WAT, would result in a higher number of adipocytes.

The excess of fat mass in TLR2^−/−^ mice may be caused by an impaired ability of skeletal muscle to use lipids as a fuel substrate and of the liver to catabolize lipids, both processes leading to a shunting of lipids to adipose tissue. However, our results from the study with microfluidic cards suggest that the loss of function of TLR2 mainly affects WAT. There may be an abnormal signal that affects adipose tissue metabolism and alters fuel partitioning in TLR2^−/−^ mice, directing increased storage in adipose tissue instead of use in muscle. The studies in WAT shed some light on the search for the mechanism: augmented Glycogen synthase 2 in the WAT of TLR2^−/−^ mice can be responsible for the increase in the storage of triglycerides in adipose tissue, initiating the cascade of events that leads to adipocyte hypertrophy [42].

Our TLR2^−/−^ mice displayed decreased glucose tolerance and increased circulating triglyceride levels, besides developing insulin resistance, which may be secondary to obesity. In this regard, TNF-α and IL-6 are elevated in TLR2^−/−^ mice that could be responsible for insulin desensitization [53,54]. High plasma NEFA levels, which we found elevated in TLR2^−/−^ mice, have been also shown to impair the actions of insulin on peripheral glucose uptake [55].

In summary, our results demonstrate a remarkable increase in fat mass due to the loss of function of TLR2 in mice and provide evidence pointing to enhanced adipogenesis when *Tlr2* is silenced. In addition, they also indicate an opposite role and a cross-regulation of TLR2 and TLR4 in adipogenesis. This work will contribute to a better understanding of the development of obesity and may help uncover new therapeutic targets to fight this disease in human patients.

## 4. Materials and Methods

### 4.1. 3T3-L1 Cell Culture and Differentiation

The mouse 3T3-L1 preadipocyte cell line (American Type Culture Collection) was cultured and differentiated according to the method published by Sadowski et al. [56].

### 4.2. Oil Red O Staining

3T3-L1 cells were fixed in buffered formalin for 1 h and stained with filtered Oil-Red O solution (Sigma-Aldrich, St. Louis, MO, USA). Stained lipid droplets within cells were imaged using a Leica microscope (Tokyo, Japan). The dye retained in the cells was eluted with isopropanol and quantified by measuring the optical absorbance at 510 nm.

### 4.3. Small Interference RNA (siRNA) Knockdown Study

Silencer Select siRNAs for mouse *Tlr2* and *Tlr4*, plus positive transfection control (*Gapdh*) and negative control (random-sequence siRNA) were purchased from Ambion Thermo Fisher Scientific (Waltham, MA, USA). 3T3-L1 preadipocytes were transfected with the corresponding silencer Select siRNAS in OptiMEM (Thermo Fisher Scientific, Waltham, MA, USA) using Invitrogen lipofectamine RNAiMax transfection agent (Thermo Fisher Scientific, Waltham, MA, USA). RNA was isolated 48 h after transfection and the levels of the targeted mRNA were monitored by RT-PCR.

### 4.4. Mice

C57Bl/6J mice were purchased from Jackson Laboratories. B6.129P2-Tlr2tm1Aki (TLR2^−/−^) and B6.129P2-Tlr4tm1Aki (TLR4^−/−^) mice were kindly provided by Dr. S. Akira (Osaka University, Japan) [57,58], and were backcrossed to the C57Bl/6J background mice for more than 10 generations. TLR2^−/−^-TLR4^−/−^ double knockout mice were generated by backcrossing TLR2^−/−^ and TLR4^−/−^ mice. Aside from breeding all colonies after purchase in our animal facilities, TLR2^−/−^, TLR4^−/−^, and TLR2^−/−^-TLR4^−/−^ double knockout mice were from time to time backcrossed to C57Bl/6J mice to renovate the colonies and maintain the genetic background. All animals were raised and maintained in a pathogen-free barrier facility with a 12-h light/dark cycle. Mice had free access to sterile water and irradiated food (standard mouse chow 2018, Kcal%: 18.6% protein, 6.2% fat, 3.5% fibre, 44.2% carbohydrate; Harlan Laboratories, Houston, TX). Body weight and food consumption per mouse were measured weekly.

### 4.5. Magnetic Resonance Studies

A total of 24 mice (WT, n = 12 and TLR2^−/−^, n = 12) divided into three groups of 3, 5, and 9 months of age were studied in a Bruker BioSpec 70/20 scanner (Bruker Biospin, Billerica, MA, USA), using a quadrature volume coil for imaging and a 1H/13C surface coil for spectroscopic studies. Animals were anesthetized with Sevofluorane, and their electrocardiograms, temperature, and respiration were monitored. A FLASH sequence was used to localize the animal, and a field map protocol was applied in a T2 weighed image with and without fat suppression. A Gaussian pulse was applied for fat suppression. For the spectroscopic studies, the coil was positioned over the abdomen of the mice, and 1H spectra were acquired. Spectra were processed with MNova software (Mestrelab Research, Santiago de Compostela, Spain).

### 4.6. Glucose and Insulin Tolerance Tests

For glucose tolerance tests, mice fasted for 16 h were injected with glucose intraperitoneally (2 g/kg body weight; Merck, Rahway, NJ, USA) and blood samples were taken at 0, 30-, 60-, 90- and 120-min. Glucose levels were measured using a glucometer (Accu-chek from Roche, Basel, Germany). For insulin tolerance tests, mice were given an intraperitoneal injection of insulin (1 IU/kg body weight; Novo Nordisk, Princeton, NJ, USA) after 4 h of fasting, and glucose levels were measured at 0, 15, 30, 60, 90, and 120 min.

### 4.7. Biochemical Analysis of Blood Samples

Serum-free insulin was assayed with an ELISA-kit (Linco Research, St Charles, MO, USA). Triglycerides and glycerol were assayed with the Triglyceride and Free Glycerol Determination kit (Sigma-Aldrich, St. Louis, MO, USA). Levels of NEFAs were determined by an enzymatic colorimetric test (FUJIFILM Wako Chemicals, Richmond, VA, USA).

### 4.8. ELISAS

Serum TNF-α, MCP-1, IL-6, leptin, and adiponectin were assayed with ELISA-based Quantikine M mouse immunoassay kits (R&D Systems, Minneapolis, MN, USA). Each sample was measured in duplicate.

### 4.9. Isolation of the Stromal Vascular Fraction from WAT

Adipose tissues were minced into fine (<10 mg) pieces in HBSS and digested for 1 h at 37 °C with 2.5 mg/ml type II collagenase (Sigma-Aldrich, St. Louis, MO, USA) in the presence of 15 mg/mL BSA (Sigma-Aldrich, St. Louis, MO, USA) and 5 mM glucose in DMEM. Adipocytes were separated from stromal vascular cells by filtration through a 250-μm nylon mesh and centrifugation for 10 min at 186× *g* [59]. The pellet comprised the SVF, and the floating cells represented the adipocyte enriched fraction. Contaminating erythrocytes were eliminated by a brief incubation in erythrocyte lysis buffer (155 mM ammonium chloride, 1 mM potassium bicarbonate, 1 mM EDTA) at 4 °C.

### 4.10. Identification of White Adipocyte Progenitor Cells

The erythrocyte-depleted SVF was depleted of endothelial cells by negative selection in an autoMACS (Miltenyi Biotec, Madrid, Spain), using CD146 mouse microbeads. The remaining cells were depleted of leukocytes by magnetic separation of the CD45-negative fraction. Positive selection of Sca-1^+^ cells was performed by using a mouse anti-Sca-1 FITC MicroBead kit (Miltenyi Biotec, Madrid, Spain). FITC-Sca-1^+^ cells were then stained with APC-anti-CD24 (BD Pharmingen, San Diego, CA, USA) and PE-anti-CD29 (eBioscience, San Diego, CA, USA). Cells were then fixed in 2% paraformaldehyde in PBS, pH 7.2. Stained cells were analyzed by flow cytometry using a FACSCanto flow cytometer (Becton Dickinson, Franklin lakes, NJ, USA), and the results were analyzed using FlowJo (Version 6.4.1; Tree Star, Ashland, OR, USA).

### 4.11. Real-Time PCR Analysis of Gene Expression

A total of 24 mice were included in the microarray expression study, four from each of the following groups: C57BL/6J and TLR2^−/−^ mice (3, 5, and 9 months of age). Total RNA was extracted from the epididymal adipose tissue with TRIzol reagent (Invitrogen; Thermo Fisher Scientific, Waltham, MA, USA). Next, 1 μg of RNA was converted to first-strand cDNA with “High-Capacity cDNA reverse transcription kit” (Applied Biosystems, Thermo Fisher Scientific, Waltham, MA, USA). The cDNA was subjected to real-time PCR by using microfluidic cards (TaqMan Low Density Arrays, Applied Biosystems, Thermo Fisher Scientific, Waltham, MA, USA) to assess the level of expression of two house-keeping genes and 93 selected genes (whose products have been involved in the development of obesity and adipocyte differentiation). LDAs were analyzed using the 7900HT system (Applied Biosystems, Thermo Fisher Scientific, Waltham, MA, USA). The data obtained from each card were analyzed using the comparative method and by normalization of expression values to 18S rRNA expression

### 4.12. Histological Analysis of Adipose Tissue

Adipose tissue was fixed in 10% buffered formalin for 24 h, dehydrated, and embedded in paraffin. Then, 5-μm sections were stained with hematoxylin and eosin. Adipocyte cross-sectional area was determined for each adipocyte in each field using ImageJ software (NIH, USA). Average adipocyte cross-sectional area was calculated using Microsoft Excel (Microsoft, Redmond,WA, USA).

### 4.13. Flow Cytometric Analysis

SVFs isolated from adipose tissue samples were centrifuged at 500× *g* for 5 min and resuspended in PBS-staining buffer at a concentration of 10 × 10^6^ cells/ml. Cells were incubated at 4 °C on a bidirectional shaker for 20 min in FcBlock (20 μg/mL) (BD Pharmingen, San Diego, CA, USA), followed by 30 additional minutes with fluorophore-conjugated primary antibodies or corresponding isotype control antibodies. Antibodies used in these studies included: F4/80-PE (2 μg/mL; eBioscience, San Diego, CA, USA). Cells were analyzed on a FACSCanto (Becton Dickinson, Franklin lakes, NJ, USA), and analysis was performed using FlowJo software (Version 6.4.1, Tree Star Inc., Ashland, OR, USA).

### 4.14. Statistical Analysis

Data are presented as mean ± SD. The data were analyzed by two factorial univariate or multivariate analyses of variance (ANOVA) or by one-way ANOVA followed by Bonferroni *t*-test (MRI analysis). *p* values less than 0.05 denote statistical significance.

## Data Availability

Not applicable.

## Supplementary Materials

The following supporting information can be downloaded at: https://www.mdpi.com/article/10.3390/ijms232415682/s1.
