# Supplementary figures and images for: Opposing Actions of TLR2 and TLR4 in Adipocyte Differentiation and Mature-Onset Obesity
Source: Int J Mol Sci. 2022 Dec 10;23(24):15682. doi: 10.3390/ijms232415682 (PMC9779340; doi:10.3390/ijms232415682)

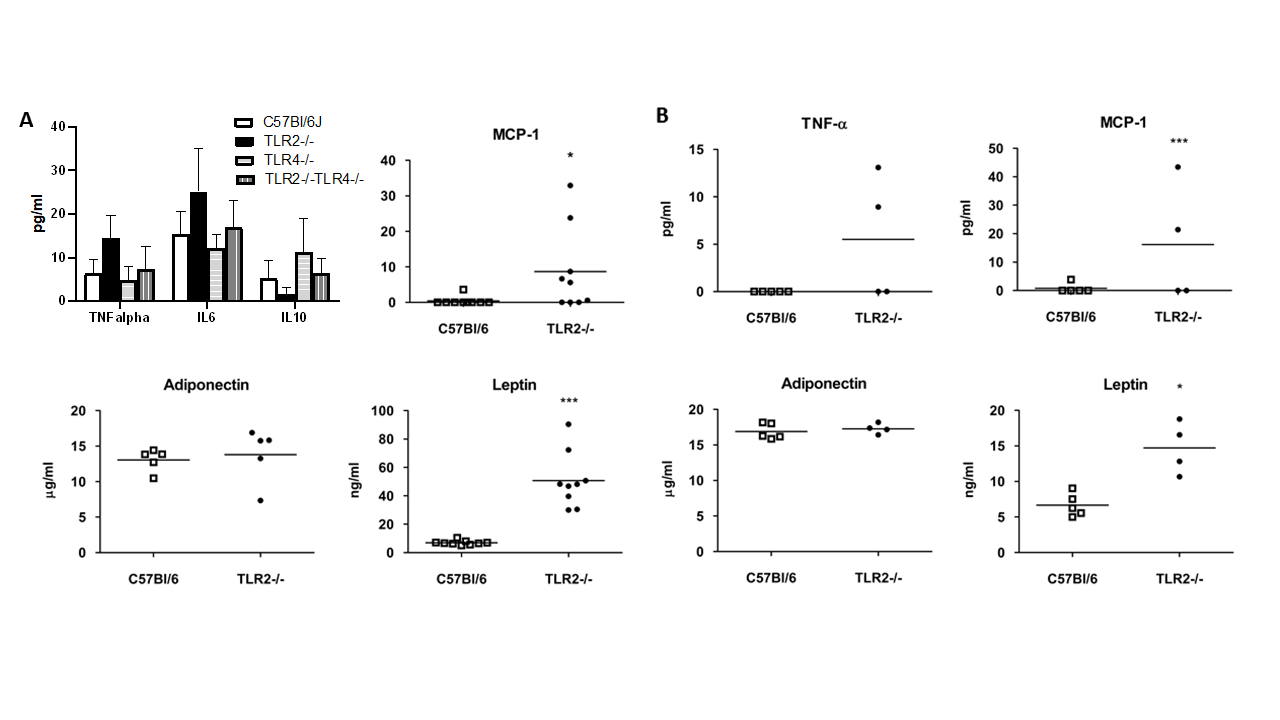

Supplement: Supplementary file 1 [file ijms-23-15682-s001.zip › Supp Fig S1.tif]

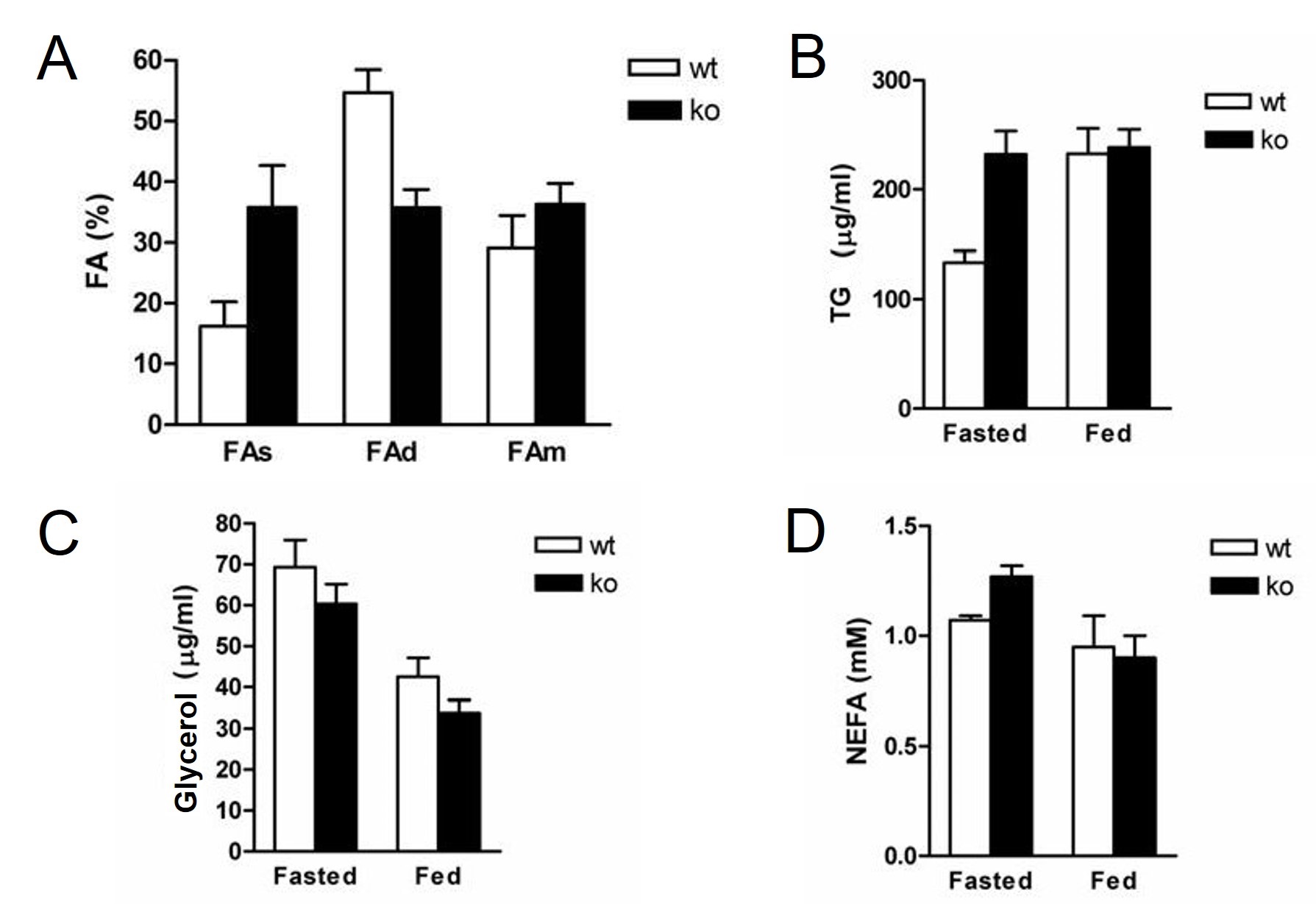

Supplement: Supplementary file 1 [file ijms-23-15682-s001.zip › Supp Fig S2.jpg]

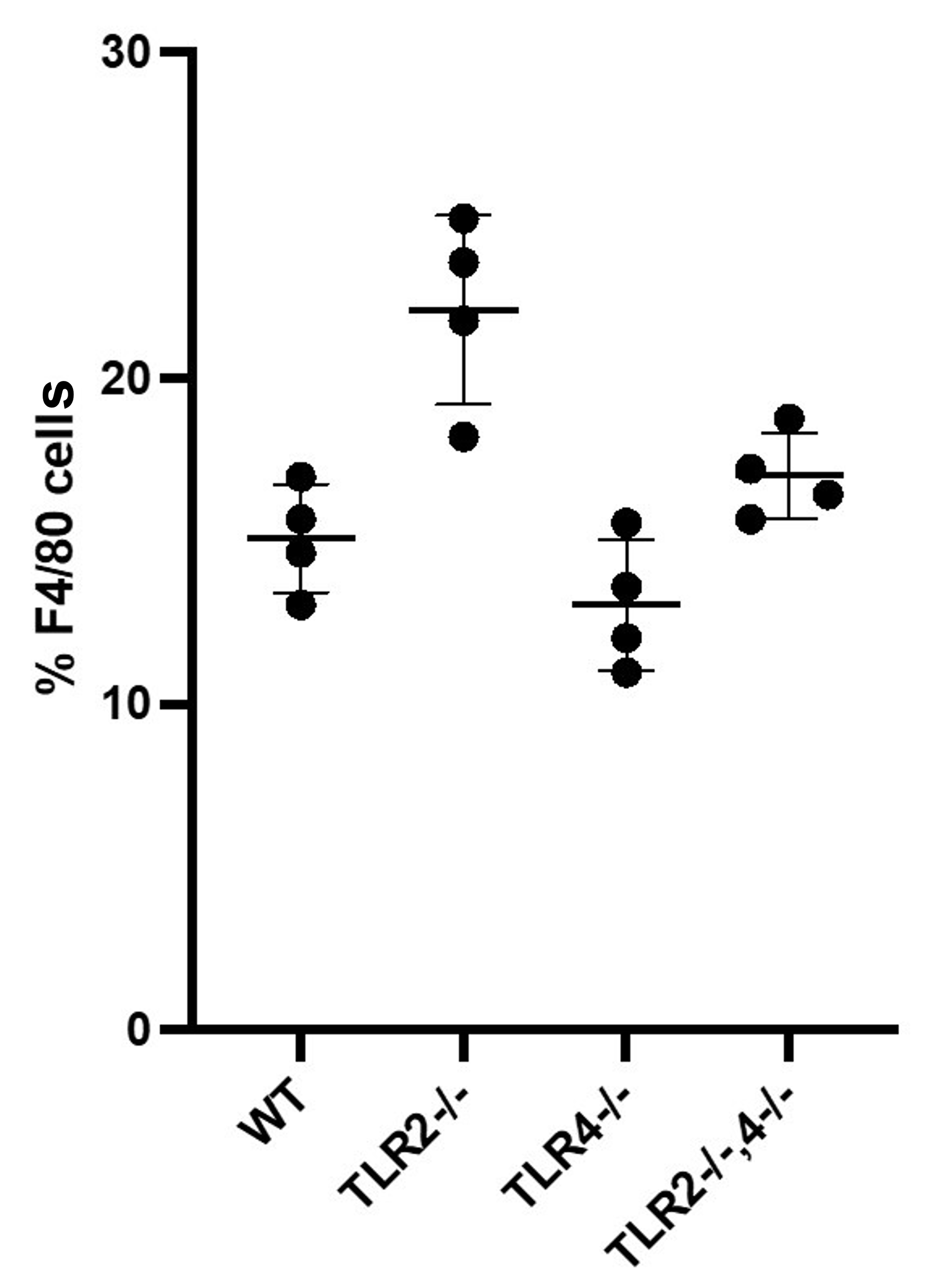

Supplement: Supplementary file 1 [file ijms-23-15682-s001.zip › Suppl Fig S3.bmp]
